# Lipid Accumulation during the Establishment of Kleptoplasty in *Elysia chlorotica*

**DOI:** 10.1371/journal.pone.0097477

**Published:** 2014-05-14

**Authors:** Karen N. Pelletreau, Andreas P. M. Weber, Katrin L. Weber, Mary E. Rumpho

**Affiliations:** 1 Department of Molecular and Cell Biology, University of Connecticut, Storrs, Connecticut, United States of America,; 2 Institute of Plant Biochemistry, Cluster of Excellence on Plant Sciences (CEPLAS), Heinrich-Heine University, Dusseldorf, Germany; Pennsylvania State University, United States of America

## Abstract

The establishment of kleptoplasty (retention of “stolen plastids”) in the digestive tissue of the sacoglossan *Elysia chlorotica* Gould was investigated using transmission electron microscopy. Cellular processes occurring during the initial exposure to plastids were observed in laboratory raised animals ranging from 1–14 days post metamorphosis (dpm). These observations revealed an abundance of lipid droplets (LDs) correlating to plastid abundance. Starvation of animals resulted in LD and plastid decay in animals <5 dpm that had not yet achieved permanent kleptoplasty. Animals allowed to feed on algal prey (*Vaucheria litorea* C. Agardh) for 7 d or greater retained stable plastids resistant to cellular breakdown. Lipid analysis of algal and animal samples supports that these accumulating LDs may be of plastid origin, as the often algal-derived 20∶5 eicosapentaenoic acid was found in high abundance in the animal tissue. Subsequent culturing of animals in dark conditions revealed a reduced ability to establish permanent kleptoplasty in the absence of photosynthetic processes, coupled with increased mortality. Together, these data support an important role of photosynthetic lipid production in establishing and stabilizing this unique animal kleptoplasty.

## Introduction

**Figure 1:**
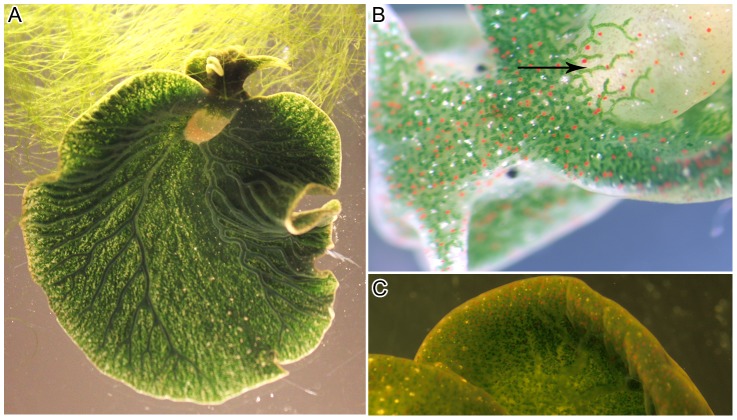
Anatomy of the sacoglossan mollusc *Elysia chlorotica*. (A) Sea slug consuming its obligate algal food *Vaucheria litorea*. Small, punctate green circles are the plastids located within the extensive digestive diverticula of the animal. (B) A defined tubule of the digestive diverticula extending into the parapodial region of the animal (arrow). The digestive system consists of densely packed tubules that branch throughout the animal's body. Each tubule is made up of a layer of single cells containing animal organelles and numerous algal plastids. This cell layer surrounds the lumen. (C) Magnified image of the epidermis of *E. chlorotica* showing densely packed plastids. The animals are light grey in color without their resident plastids, which contribute chlorophyll to render the sea slugs bright green.

The sacoglossan marine mollusc *Elysia chlorotica* Gould exhibits a unique symbiotic relationship with its algal food *Vaucheria litorea* C. Agardh reviewed by [1]–[3]. In this symbiosis, only the plastids ( = chloroplasts) of the algal food are sequestered by the sea slug host, no other algal organelles are retained, and the term kleptoplasty (retention of “stolen plastids”) is used to define the plastid symbiosis [4]. Once the *V. litorea* plastids are ingested by the host, they are incorporated intracellularly into the cells lining the highly branched digestive diverticula of the animal (Figure 1). Numerous plastids reside within the digestive cells, and they continue to photosynthesize for several months in the animal [5], [6]. It has been speculated that the plastids avoid damage in the lumen of the digestive diverticula due to the presumed mild nature of the *E. chlorotica* digestive enzymes, modified for digesting cell sap [7]. Additionally, it is likely that the plastids of this coenocytic alga are more robust and may withstand the mechanical stress of ingestion better than plastids of other algal species [8], [9]. However, once inside the animal cells, the plastids must still avoid detection by *Elysia* and subsequent degradation. Plastid division has not been observed in the animals; this is likely due to the lack of the algal nucleus and requisite replication machinery. Yet, animals collected from the wild and subsequently starved in the laboratory (provided with only light and CO_2_) can be sustained for up to 10 months with no additional food [2], [3], [5], [6], [10]. Although this kleptoplasty was first described nearly 50 years ago [11], [12], the mechanisms underlying plastid function in the foreign animal cell remain unclear.

Another long-standing mystery surrounding this association concerns the mechanisms involved in the initial establishment and stabilization of the plastids in the animal. Historical work on the *Elysia-Vaucheria* symbiosis has focused on wild-collected adult animals. Recent work in our laboratory provided a continuous culture system in which we were able to observe and characterize the actual establishment of the symbiosis immediately following metamorphosis of the veliger larvae into juvenile sea slugs [13]. Investigating this particular time-frame of the life history may help unravel how the symbiosis is established. It appears there is a distinct period of time required for plastid stabilization in the host tissue of newly developed juveniles. If newly metamorphosed juvenile animals are allowed to feed on *Vaucheria* for 7 d (or longer), plastids remain stable in the animal tissue even upon subsequent removal of food [13]. The juveniles enter into “permanent” kleptoplasty and can sustain long periods (up to 4 wk) of subsequent starvation. If, however, the juvenile animals are removed from food prior to feeding for 7 d, the plastids are quickly broken down and the animals cease to develop. We refer to this latter period in juvenile animals as “transient” kleptoplasty wherein the plastids are intracellular, but still subject to breakdown by the host. Although we have repeatedly observed this phenomenon at the macro level, the underlying cellular processes and mechanisms remain to be explored. This study set out to address the mechanisms involved in the establishment of permanent kleptoplasty at the cellular level during the initial development of post-metamorphic *E. chlorotica*.

## Methods

### Culturing of *E. chlorotica* for Electron Microscopy

*Elysia chlorotica* juvenile animals, defined as 1–14 days post metamorphosis (dpm), were cultured in the laboratory following methods outlined in Pelletreau et al. [13]. Animals were originally collected from salt marshes located in Martha's Vineyard, MA, USA (*E. chlorotica* does not fall under endangered or protected status and no specific permissions were required for collections of *E. chlorotica* from the field). For this study, animals from one brood of eggs were divided into individual wells of a multiwell plate with 4 ml artificial seawater. *Vaucheria litorea* was added to enable feeding and plastid sequestration. Newly metamorphosed animals were aged by the date of metamorphosis and secondarily upon key developmental features based on the extent and pattern of chloroplast distribution within the animal's digestive diverticula [13]. Three replicate animals for each time point were fixed, although only one animal for each time point was visualized using transmission electron microscopy (TEM). Control animals were fed *V. litorea* daily for 1–14 dpm and fixed on daily intervals (n = 3 per time point). Transient kleptoplasty was represented by animals fed *V. litorea* for 5 d, and then starved for 1–7 days post feed (dpf). Permanent kleptoplasty was represented by animals fed *V. litorea* for 7 d and then starved for 1–7 dpf. Two additional comparative treatments were used: aposymbiotic animals and mature adults. Aposymbiotic animals were 1 dpm animals that were induced to metamorphose using *V. litorea* contained within dialysis tubing. This allowed for the yet unknown chemical inducer of metamorphosis to affect the competent veligers, but prevented the metamorphosed animals from feeding on *V. litorea*. Hence, these aposymbiotic animals were 1 dpm, but had no ingested *V. litorea* chloroplasts. Additionally, sexually mature adult (∼11 months) *E. chlorotica* specimens that had been continually fed in the laboratory were visualized and compared to adults of the same age and feeding history that had been subsequently starved for 2 wk. These animals are referred to as “adult” specimens throughout; all others are juveniles (14 dpm or younger as specified) or aposymbionts.

At the appropriate time point, *E. chlorotica* specimens were fixed on ice in 1% (v/v) gluteraldehyde and 4% (v/v) formaldehyde in 0.15 M sodium cacodylate buffer (0.58 M sucrose; pH 7.5) and stored overnight at 4°C. Samples were rinsed 3× in 0.15 M sodium cacodylate buffer before post-fixation in 2% osmium tetroxide for 1 h, followed by three rinses with distilled water. Samples were brought through a dehydration series of 50-70-95-100% acetone with three 7-min rinses per acetone concentration. The 50 and 70% acetone rinses were done on ice and the remainder at room temperature. After the final dehydration step, samples were infiltrated with a 50∶50 mixture of 100% acetone/Epon-Araldite resin and allowed to sit overnight at room temperature. Samples were transferred through two fresh aliquots of the Epon-Araldite mixture and placed under vacuum for 5 min before embedding in the Epon-Araldite mixture in blocks with each specimen oriented identically. Blocks were cured for 48 h at 65°C. Once cured, samples were faced and trimmed with every effort towards capturing the same region of the individuals within the block face. To maintain consistency between samples, the region between the initiation of the parapodia and the eye spots was targeted for the block face. Thick sections (0.75 µm) were cut using a glass knife on a Sorval JB4 microtome, and the sections stained using toluidine blue. Thick sections were inspected using light microscopy for orientation prior to ultrathin sectioning for TEM. Thin sections (40–90 nm) were cut with a Leica EM UC6 ultramicrotome using a diamond knife. Sections were captured on copper grids and stained with 2% uranyl acetate for 30 min and subsequently with 0.5% lead citrate for 5 min. Sections were visualized on a Phillips/FEI CM 10 electron microscope. Micrographs (n = 3 per treatment) were quantified using ImageJ software (http://rsb.info.nih.gov/ij/) and the percent by area of each image that was either lipid or plastid was calculated and compared using a Welch's *t* test.

### Confocal Microscopy

Lipid composition was verified using confocal microscopy and BODIPY © 505/515 (4,4-difluoro-1,3,5,7-tetramethyl-4bota-3a,4a-diaza-s-indacene), a neutral lipid stain superior to Nile Red in certain systems [14], [15]. To verify that bodies observed in both light and electron microscopy were in fact lipid droplets (LDs), digestive diverticula were dissected from adult *E. chlorotica* tissue, stained with 1 µg ml^−1^ final concentration of BODIPY 505/515, and visualized on a Nikon A1R confocal microscope. Excitation/emission wavelengths (488 and 515 nm) were used to detect BODIPY labeled lipids while plastid chlorophyll autofluorescence was detected using a 680 nm emission filter. This adult specimen had been starved for 1 month prior to visualization. Spectral detection was performed on the presumed LDs to confirm the observed fluorescence was due to BODIPY 505/515 labeling and not to potential autofluorescence.

### Fatty Acid Analysis

Adult *E. chlorotica* specimens reared in the laboratory and fed *V. litorea* twice weekly were used for lipid analysis. To investigate the short-term effects of food removal, these mature, fed adults were then divided into two groups: 1) animals provided *V. litorea* daily for 14 d (fed), and 2) animals starved of food for the same period of time (starved). *V. litorea* was obtained from a unialgal culture maintained in our laboratory. Wet weights of blotted specimens were recorded prior to freezing in liquid nitrogen and shipping on dry ice to the Institute of Plant Biochemistry at Heinrich-Heine University for fatty acid analysis.

Samples (10–15 mg FW) were freeze-dried overnight before extracting with 2 ml chloroform containing the internal standard 17∶0 FFA (25 µg/ml) and 1 ml methanol. Subsequently, 1 ml 0.9% (m/v) sodium chloride was added. Samples were centrifuged, and 0.5 ml of the chloroform phase was removed and dried under a stream of nitrogen gas.

The dried residues were resolved in 1 ml 3N HCl/methanol and incubated for 1 h at 90°C. After cooling to room temperature, 1 ml hexane and 1 ml 0.9% sodium chloride were added. Samples were centrifuged and 1 µl of the hexane phase was injected into the GC/MS. Fatty acid methyl esters (FAMES) were separated on an Agilent HP5 – MS column (30 m length, inner diameter 0.25 mm, Film 0.25 µM), using the following temperature program: 70°C hold for 1 min, ramp to 170°C with steps of 25°C/min, ramp to 220°C, with steps of 3°C/min, ramp to 300°C with steps of 25°C/min, hold for 10 min. FAMES were detected using a Waters-Micromass GCT Premier time-of-flight mass spectrometer, using electron impact ionization (source temperature 200°C, electron impact energy 70 eV, trap current 200 µA). Qualitative and quantitative data analysis was conducted using the Waters MassLynx software package. Fatty acid contents are reported relative to the internal standard (FFA 17∶0), normalized to fresh weight of the sample.

### Plastid Establishment in Light/Dark Conditions

*E. chlorotica* were allowed to establish kleptoplasty under various light conditions to test if photosynthetic processes and/or putative LDs generated via photosynthesis, were involved in establishment or permanency of the symbiosis. Newly metamorphosed animals from the same brood (≤4 dpm; permanent kleptoplasty had not been established) were placed in individual wells containing 5 ml of 0.2 µm filtered artificial seawater with a salinity of 32 psu. Animals were maintained at 24°C in 24 h light (24L), 12L∶12D, or 24 h dark (24D) conditions (n = 24 animals each) and subsequent growth and plastid establishment observed. Light intensities were 25 µE m^−2^ s^−1^. For the first 4 wk of the experiment, animals were fed *V. litorea* twice weekly to enable the establishment of kleptoplasty. After 4 wk of being fed, the animals were subsequently starved, yet maintained in the same light conditions. Water changes were done weekly. Photographs were taken after the first and fourth week of feeding and 4, 6, and 8 wk after subsequent starvation. Size was quantified using ImageJ software (http://rsb.info.nih.gov/ij/) and pigment color observed as a proxy of plastid presence/loss. Plastid loss in each animal after 4 wk starvation was assessed using the following metric: 1 =  dark green (no change in color); 2 =  light green in color, 3 =  yellow to brown in color, or 4 =  no chlorophyll pigment observed.

### Analysis

Image labeling, orientation, cropping, and brightness-contrast adjustments were done using Adobe Photoshop CS5. A Welch's *t*-test was used to determine significant differences in the area of lipids and plastids in electron micrographs (presented as mean ± SD). Analysis of Variance (ANOVA) was used to determine significant differences in lipid composition (presented as mean ± SE) between algae, fed animals and starved animals. Changes in size (presented as mean ± SE) of *E. chlorotica* were analyzed with Analysis of Variance Repeated Measures (ANOVAR) after testing for Sphericity using a Mauchly's test. Due to high mortality rates in the 24D treatment, the three treatment groups had unequal sample sizes. To account for this and enable statistical analysis, the data were normalized to the sample size remaining at the end of the experiment (n = 9). In time points with >9 replicates per treatment remaining, 9 animals were randomly selected using a random number generator and these data presented. Significance was defined as p<0.05. Post-hoc Tukey's HSD tests were done to determine which treatments were significantly different from each other. Statistics were performed using SPSS V. All graphics were made using SigmaPlot 2000 or Microsoft Excel.

## Results

### General observations of developing *Elysia chlorotica*

**Figure 2:**
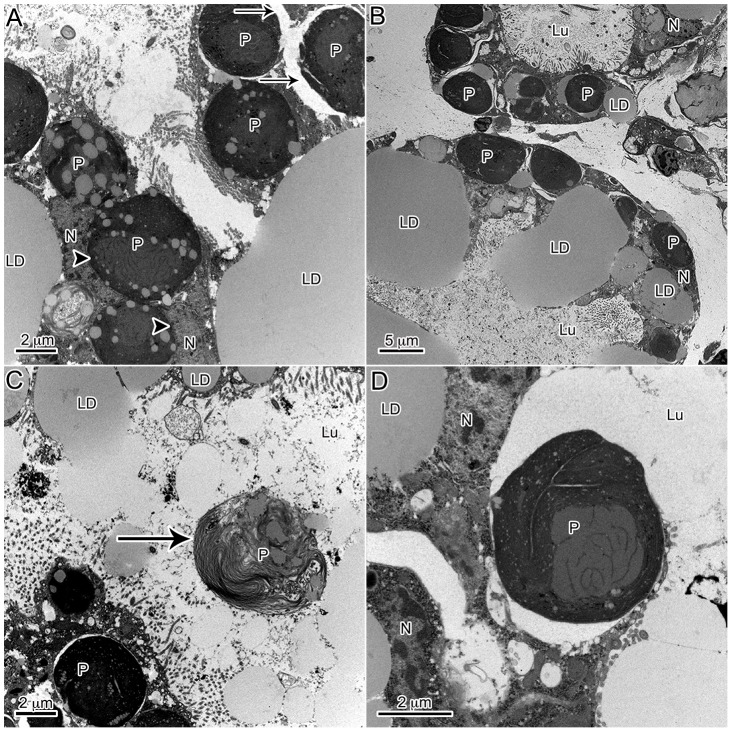
General observations of plastid dynamics within the digestive diverticula of *E. chlorotica* developing juveniles (<9 dpm). Many cellular observations in juveniles were in agreement with historical work on adult *E. chlorotica*, with the notable exception of massive LD accumulation in juvenile animals. (A) Numerous plastids accumulated within the cells of the digestive diverticula both surrounded by a membrane (arrow) or in apparent direct contact with the host cytosol (arrowhead). (B) Accumulation of the plastids corresponded with lipids, while the lumen of the animals remained relatively clear of debris. (C) A rare degraded plastid (arrow) observed within the lumen. (D) One rare example of potential phagocytosis of an algal plastid by the digestive cells. LD: lipid droplet, P: plastid, Lu: lumen, N: *E. chlorotica* nucleus.

Several microscopic studies of kleptoplastic *Elysia* have characterized the distribution and location of plastids in adult tissue [16]–[18]. Here, developing *E. chlorotica* in the process of establishing permanent kleptoplasty have been described, and many similar trends were observed. Similar to previous reports of adult *E. chlorotica*, numerous plastids were observed within the digestive cells of the developing juveniles. At times, these plastids appeared in direct contact with the cytosol or, conversely, surrounded by a membrane, presumably host derived (Figure 2A). In general, there was very little “debris” observed in the digestive lumen of the animals, algal or otherwise, including a surprising lack of any obvious microbial community (Figure 2B). Only on rare occasions were degraded plastids observed in the lumen of the animal (Figure 2C). Although numerous sections were observed from various feeding states (recently fed to weeks starved), in only one image was there the suggestion of possible phagocytosis (Figure 2D). One observation distinct from historical work with adult tissue, and that elicited further exploration (below), was the overwhelming abundance of accumulated lipids within the developing *E. chlorotica* digestive tissue.

### Presence of lipid droplets (LDs) in *Elysia chlorotica*

**Figure 3:**
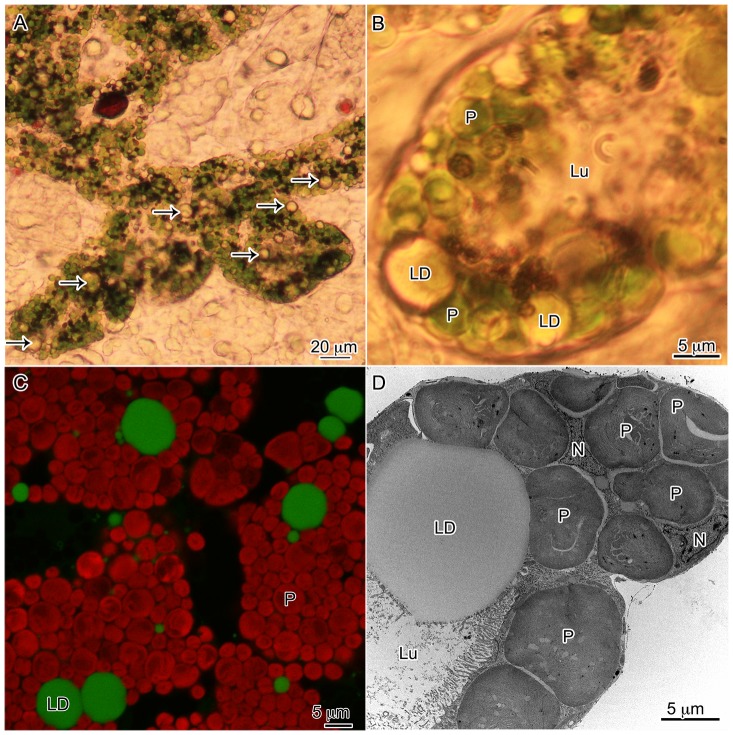
Lipid droplets in the digestive diverticula of *E. chlorotica* as observed using light (A,B) confocal (C) and transmission electron (D) microscopy. Green fluorescence in panel C is from the neutral lipid fluorescent label BODIPY 505/515 (Molecular Probes) verifying these abundant refractive bodies as lipids, and the red is chlorophyll autofluorescence. Animals in these image range in age from 4 dpm (A,B) to >6 months (C,D). Arrows (A) and LD: lipid droplet, P: plastid, Lu: lumen, N: *E. chlorotica* nucleus.

The LDs observed in this study exhibited a unique staining pattern observed with TEM. Whereas lipids often stain black when using OsO_4_ as a post-stain, the lipids in *E. chlorotica* appeared to be less optically dense. However, observations using light and confocal microscopy confirmed that the large bodies repeatedly seen were comprised of lipids (Figure 3). The fluorescent probe BODIPY 505/515, specific for neutral lipids, clearly bound to the refractive bodies observed within the digestive diverticula (Figure 3C). The LDs were often closely associated with plastids within the animal, and were present in varying sizes throughout the digestive tubules (Figure 3A,B). It was often observed that the plastids formed a tightly associated ring around a large central LD localized at the terminal end of a digestive tubule (Figure 3B,D).

### Plastid dynamics, LD emergence and establishment of permanent kleptoplasty

**Figure 4:**
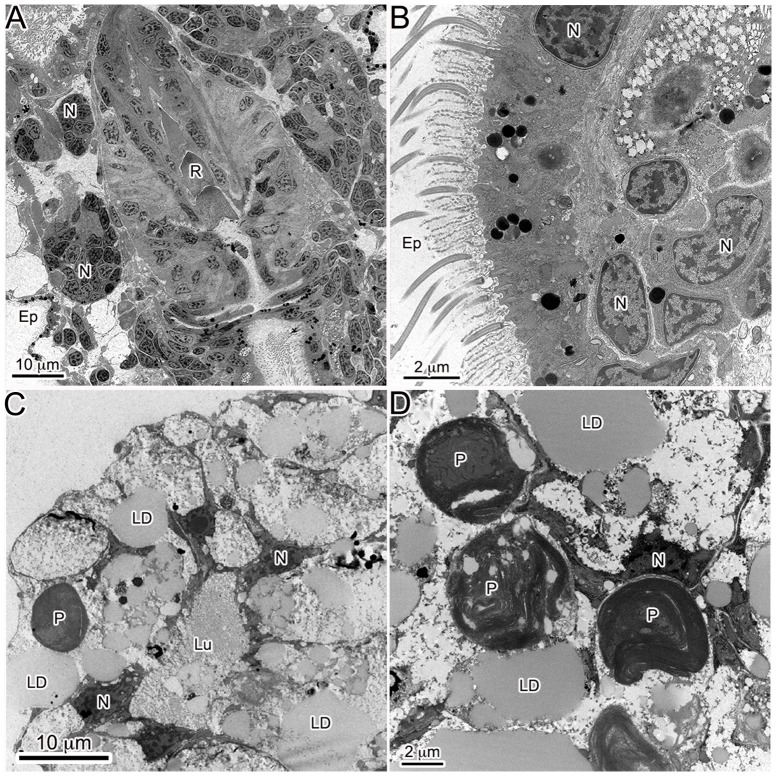
Transmission electron micrographs of *E. chlorotica* illustrating the acquisition of both lipids and plastids upon feeding. (A,B) Aposymbiotic *E. chlorotica* at 1 dpm with neither plastids or lipids present in the digestive tissue. (C,D) Fed *E. chlorotica* at 1 dpm with large lipid accumulation and visible intracellular plastids. LD: lipid droplet, P: plastid, Lu: lumen, N: *E. chlorotica* nucleus, Ep: epidermis, R: radula.

**Figure 5:**
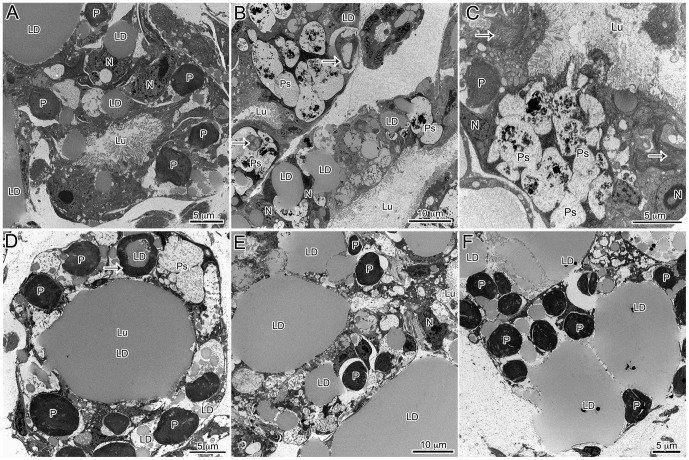
Representative images of the digestive diverticula of juvenile *E. chlorotica* allowed to feed on *V. litorea* for 5 days prior to being starved of food ( =  transient kleptoplasty). Animals were starved for 1 day (A), 3 days (B) or 7 days (C). The same age control animals, allowed to feed continuously, are in the corresponding position in the bottom panels (D–F). Plastid decay and LD degradation are apparent in the animals removed from food in comparison to the same age controls. LD: Lipid droplet, P: plastid, Lu: lumen, N: *E. chlorotica* nucleus, Ps: phagosome; arrows indicate decaying plastids, black marks in F are staining artifacts.

**Figure 6:**
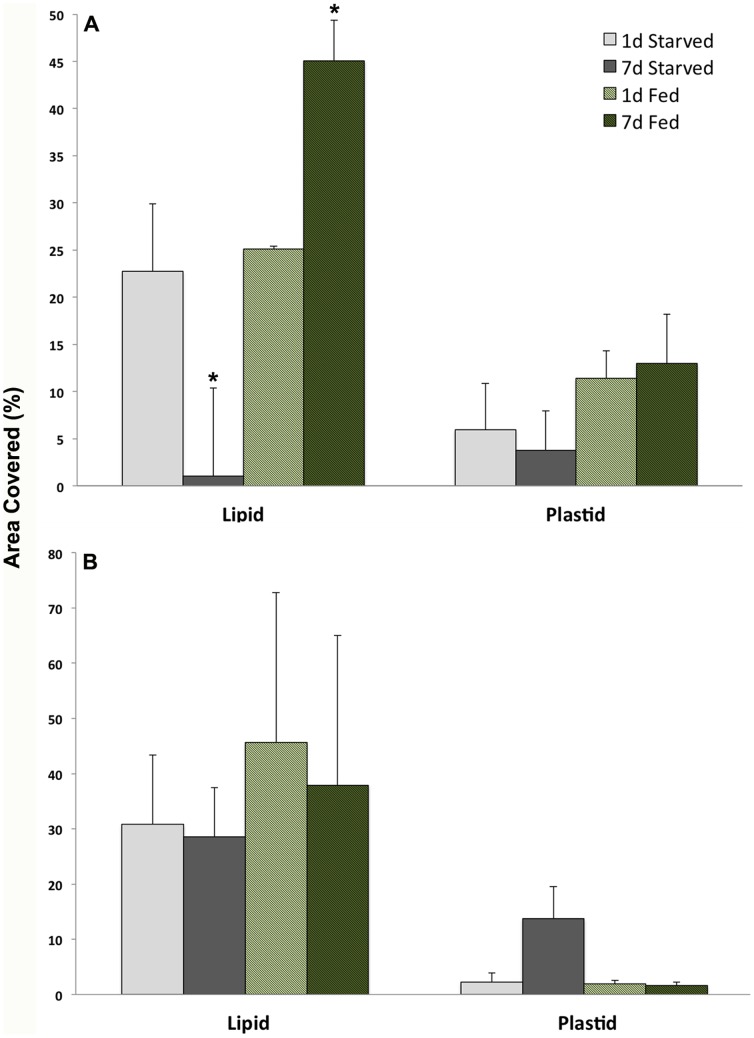
Differences in the percent cover of lipids and plastids based on area in TEMs of animals exhibiting transient and permanent kleptoplasty. (A) Percent cover (mean ± SD) of lipids and plastids from micrographs (n = 3) of animals fed 5 d (transient kleptoplasty) and then starved for 1 or 7 d (grey bars) compared to same age control animals fed continuously (green bars). (B) Percent cover (mean ± SD) of lipids and plastids from micrographs (n = 3) of animals fed 7 d (permanent kleptoplasty) and then starved for 1 or 7 d (grey bars) compared to control animals the same age but fed continuously (green bars). Asterisks indicate significantly different means (Welch's *t*-test; p = 0.05).

**Figure 7:**
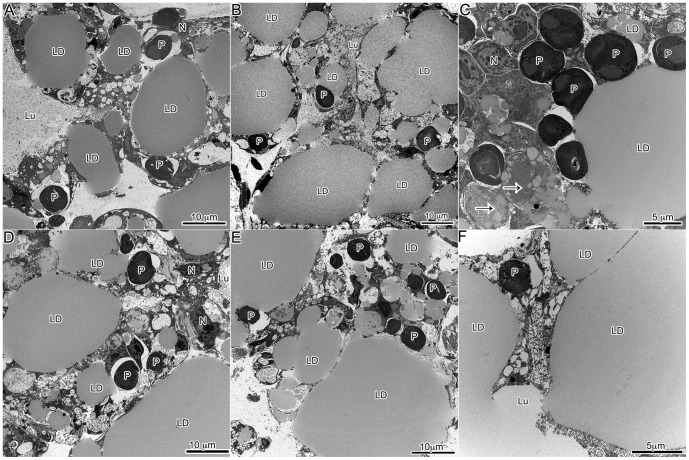
Representative images of the digestive diverticula of juvenile *E. chlorotica* allowed to feed on *V. litorea* for 7 days prior to being starved of food ( =  permanent kleptoplasty). Animals were starved for 1(A), 3 d (B) or 7 d (C). The same age control animals, allowed to feed continuously, are in the corresponding position in the bottom panels (D–F). Starved and fed animals show very similar patterns. LD: lipid droplet, P: plastid, Lu: lumen, N: *E. chlorotica* nucleus; arrows indicate decaying plastids.

TEM observations of developing juveniles showed a clear relationship between lipid production and plastid incorporation. Aposymbiotic animals that had no physical contact with *V. litorea* showed no accumulation of lipids (Figure 4A,B), but within 24 h of feeding, both plastids and lipids were observed in the animal (Figure 4C,D), and both continued to accumulate as long as *V. litorea* was available to the animal. *E. chlorotica* juveniles allowed to feed for 5 d before starvation ( =  transient kleptoplasty) showed a rapid degradation of both plastids and lipids within 24 h of food removal (Figure 4A), becoming even more apparent after 3 and 7 d of starvation (Figure 5B,C). Virtually no intact plastids or LDs were observed in the digestive cells after one week of starvation ( =  12 dpm animals, Figure 5C) relative to control animals fed daily for 12 d (Figure 5F). The difference in the percent by area of each micrograph represented by lipids was significantly lower after 7 d of starvation (Figure 6A). Phagosomes appeared more numerous in starved animals (Figure 5C). In stark contrast, animals allowed to feed for 7 d before being starved an additional 7 d showed no change in internal plastid distribution or LD accumulation relative to same age controls (Figures 6B, 7).

**Figure 8:**
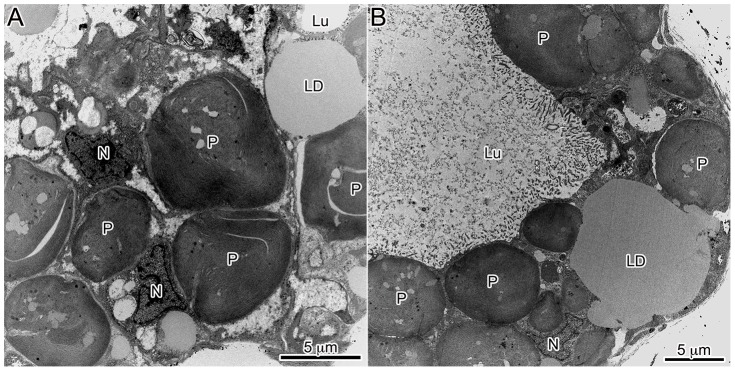
Adult *E. chlorotica* (reared in the laboratory for 11 months and fed *V. litorea*) were used to compare the effects of food removal on the digestive diverticula of mature individuals. Starvation of adult animals showed no obvious reduction in lipid or plastid content. (A) Adult animal starved for 2 wk. (B) Adult animal fed continuously. LD: lipid droplet, P: plastid, Lu: lumen, N: *E. chlorotica* nucleus.

To investigate the effect of starvation on plastids and LDs in adult animals, 11 month old *E. chlorotica* reared and fed in the lab were used for comparison. Adult *E. chlorotica* specimens starved for a 2 wk period were compared to continuously fed adults from the same brood using TEM. No clearly observable differences in either plastid or LD pattern were noted between the starved vs. fed adult animals regardless of recent feeding history (Figure 8A,B). Plastids and LDs in starved adult animals were numerous, and there were no regions of intense plastid decay (Figure 8A), as had been seen in juveniles removed from food before permanent kleptoplasty had been acquired (Figure 5A–C). The visible effects of food removal at the cellular level, represented by plastid and LD decay and increased phagosomes, were only observed when animals were denied food prior to 7 d of age (Figure 5), and not in juveniles fed >7 d (Figure 7), or in adults (Figure 8).

### Putative source of LDs

**Figure 9:**
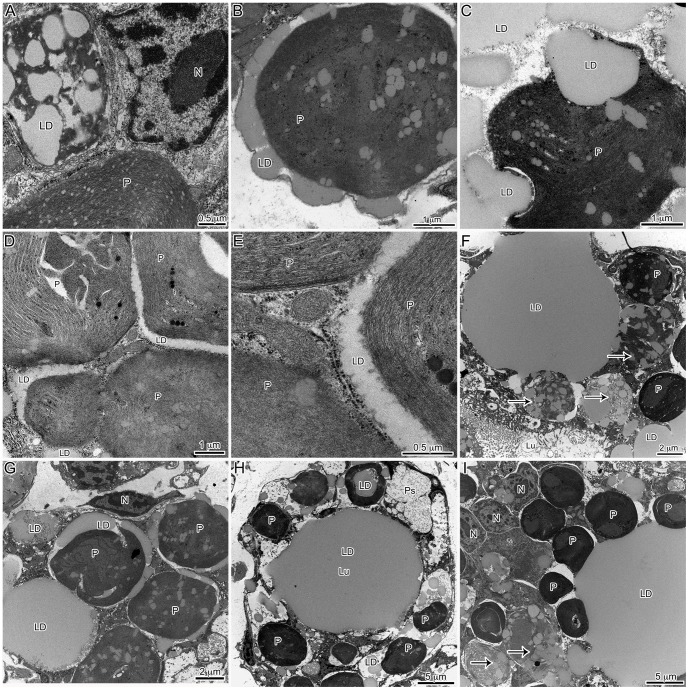
Representative TEM images of LD dynamics during the initial 2*E. chlorotica* suggesting the source of lipid accumulation in the animal is the algal plastids. All images are of fed controls. Accumulation of LD within the plastids is visible in every panel, but most clearly in A–C. Exudation of lipids from the plastids is suggested by panels B–G. Accumulation of large intracellular LD is illustrated in F and G, and intra-lumenal LD in H and I. LD: lipid droplet, P: plastid, Lu: lumen, N: *E. chlorotica* nucleus; arrows indicate decaying plastids.

Repeated observations of plastids within the developing animals showed several trends that may help deduce a functional role of the LDs during plastid establishment. Often, accumulation of LDs was observed *within* the plastids (Figure 9A,B,C,F,G). Intracellularly, plastids were often tightly surrounded by lipids and both appeared to be bound by a membrane (Figure 9B,C,D,E,G). Small LDs were exuded from plastid membranes (Figure 9B–E,G) and subsequently accumulated into large adjacent LDs (Figure 9F,G). Exudation was apparent from intact as well as degraded plastids (Figure 9G,I). Lipids accumulated, often larger in size than the plastids, and were arranged in close association with numerous surrounding plastids (Figure 9F,I). At times, lipids completely filled the lumen of the digestive diverticula (Figure 9H,I). These images suggest that the source of the LDs in the animal is the plastids themselves. It is not possible to say if the plastids are producing free fatty acids that may then be further metabolized by the animals, or if they are exuding triacylglycerols (TAGs) directly into the animal tissue.

### Fatty acid analysis

**Figure 10:**
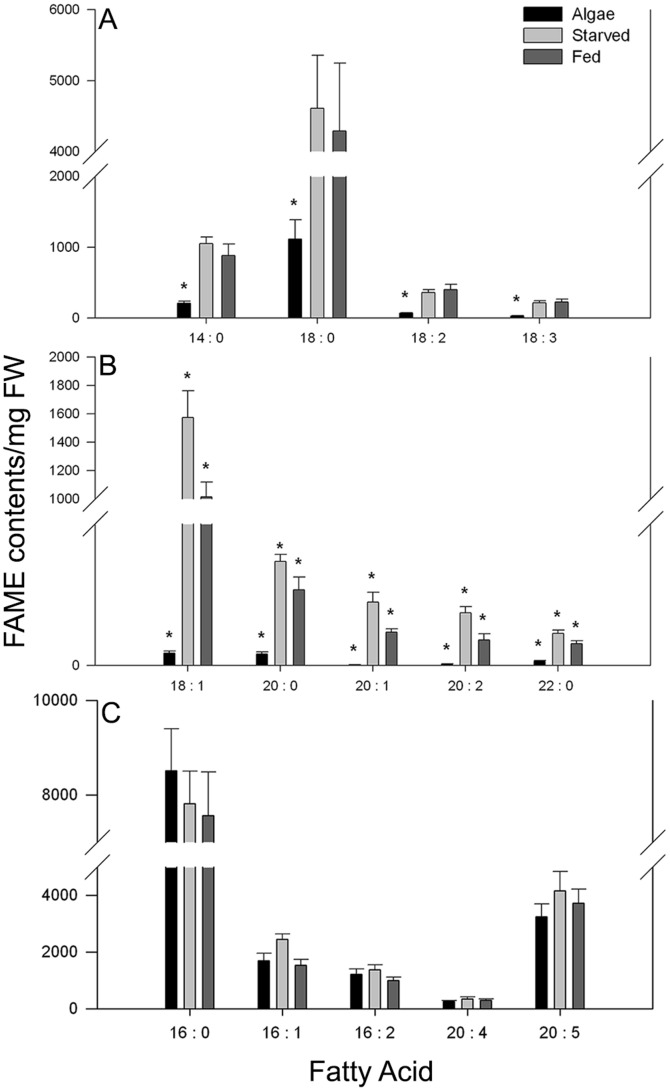
Fatty acid profile of *V. litorea* (alga) and adult *E. chlorotica* fed regularly (Fed) or starved for 2 wk (Starved) as determined by FAME analysis (FAME contents/mg FW, normalized to 17∶0 internal standard). (A) Fatty acids that were significantly lower in the alga than in either fed or starved animals. (B) Fatty acids that were significantly variable between all three samples. (C) Fatty acids that showed no significant difference between algal and animal samples. Asterisk indicate significance; ANOVA p<0.05.

An initial comparison of the fatty acid moieties (after lipid hydrolysis and transesterification) found in *V. litorea* and adult *E. chlorotica* (fed continuously or starved 14 d; comparable to the adults visualized in Figure 8) revealed several candidate fatty acids that may be generated by the plastids. Of the 14 fatty acids identified in the analysis, four (14∶0, 18∶0, 18∶2, 18∶3) showed significantly lower amounts in *V. litorea* when compared to either fed or starved animals (Figure 10A; ANOVA p<0.05). In a second subset of fatty acids (18∶1, 20∶0, 20∶1, 20∶2, 22∶0), the levels in the animals were significantly greater than in the alga, and there was a concurrent significant drop in the amount of these fatty acids in starved animals relative to fed (Figure 10B; ANOVA p<0.05). In the final group of fatty acids (16∶0, 16∶1, 16∶2, 20∶4, 20∶5) there was no significant difference in the amount of each moiety in the alga or in the animals (fed or starved; Figure 10C). Only in this final group of fatty acids were the concentrations in the alga comparable to those in the animals. Because of the high concentration in the algal tissue, these fatty acids may be potential candidates for plastid derived lipids observed in the animal digestive tissue, though further experimentation is needed to confirm this.

### Kleptoplasty establishment in light vs. dark conditions

**Figure 11:**
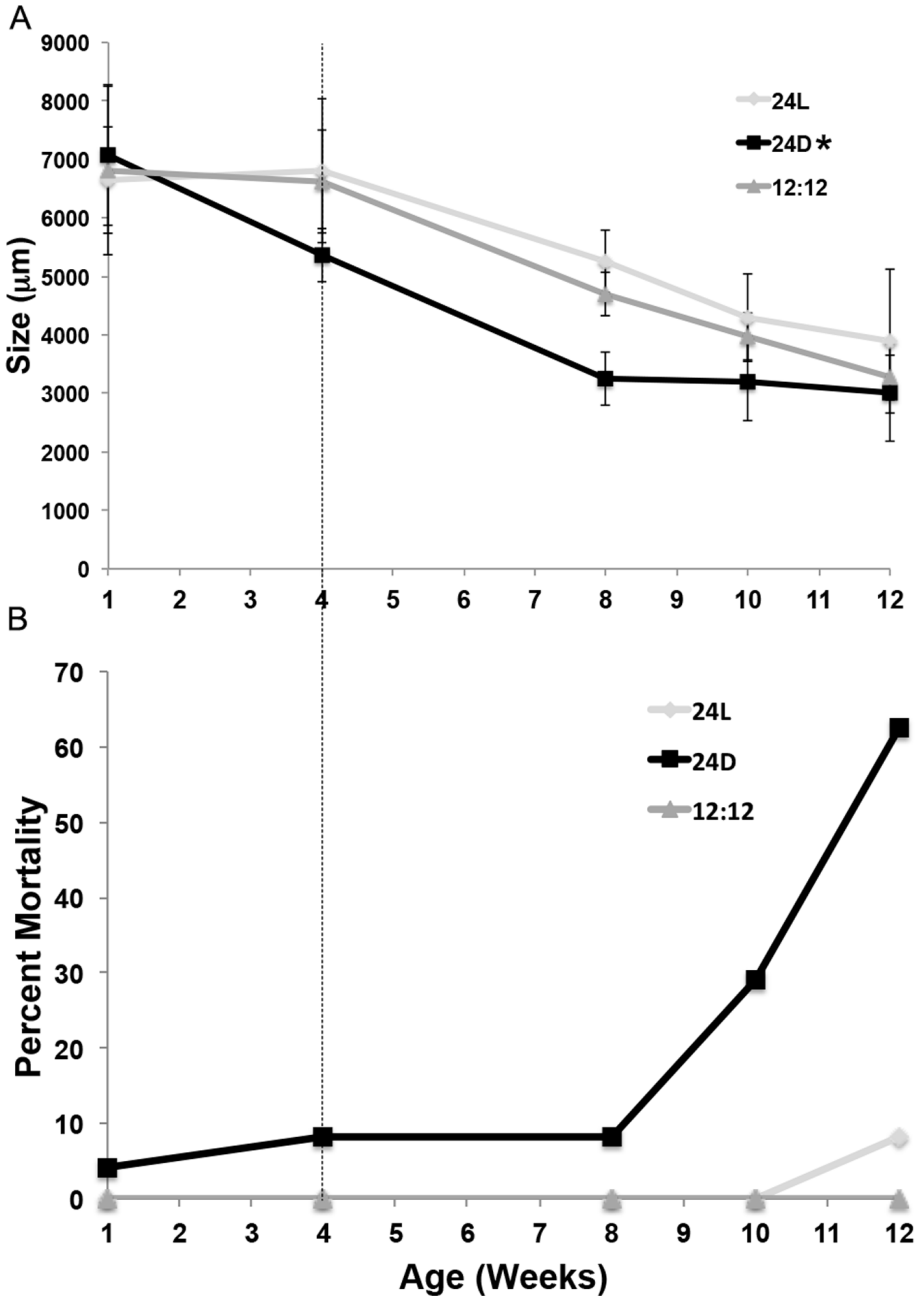
Growth rate (A) and mortality (B) of animals raised in either 24L, 12L∶12D or 24D conditions and provided *V. litorea* for 4 wk before starvation (indicated by the dotted line). (A) Mean length (± SE) of animals (n = 9, representing those animals still viable after 12 wk of starvation) after 4 wk of feeding and then 4, 6 and 8 wk of subsequent starvation in the light conditions indicated. Asterisk indicates a significant effect of treatment over time in animals starved in dark conditions relative to animals starved in the light (ANOVAR, Huyn-Feldt correction p = 0.005). (B) Percent mortality observed in the three treatment groups (n = 24, representing all replicates) over the course of the 12 wk. Mortality in the 24D reared animals was greater than in the other two treatments.

Maintaining juvenile animals in varied light conditions during the establishment phase of kleptoplasty is a simple way to regulate the production of photosynthate and, presumably, any related lipid end products. Although the animals in the three light conditions were initially the same size, there was a rapid and significant loss of size in the animals maintained in the dark, even during the initial month of feeding (Figure 11A). This loss was not due to lack of feeding by the animals in dark conditions, as the consumption of the algae was obvious (Pelletreau, personal observations). Subsequent starvation of animals resulted in a reduction of size in all three light conditions; however, the 24D treatment was significantly different than the 24L and 12L∶12D (ANOVAR using Huyn-Feldt correction p = 0.005; Figure 11A). In addition, the mortality rate of animals in the 24D treatment was near 60% after 8 wk of starvation, compared to 0% and 5% mortality in the 12L∶12D and 24L conditions, respectively. In the 24D treatment, mortality was observed at low levels before exponentially increasing after 8 wk starvation (Figure 11B).

**Figure 12:**
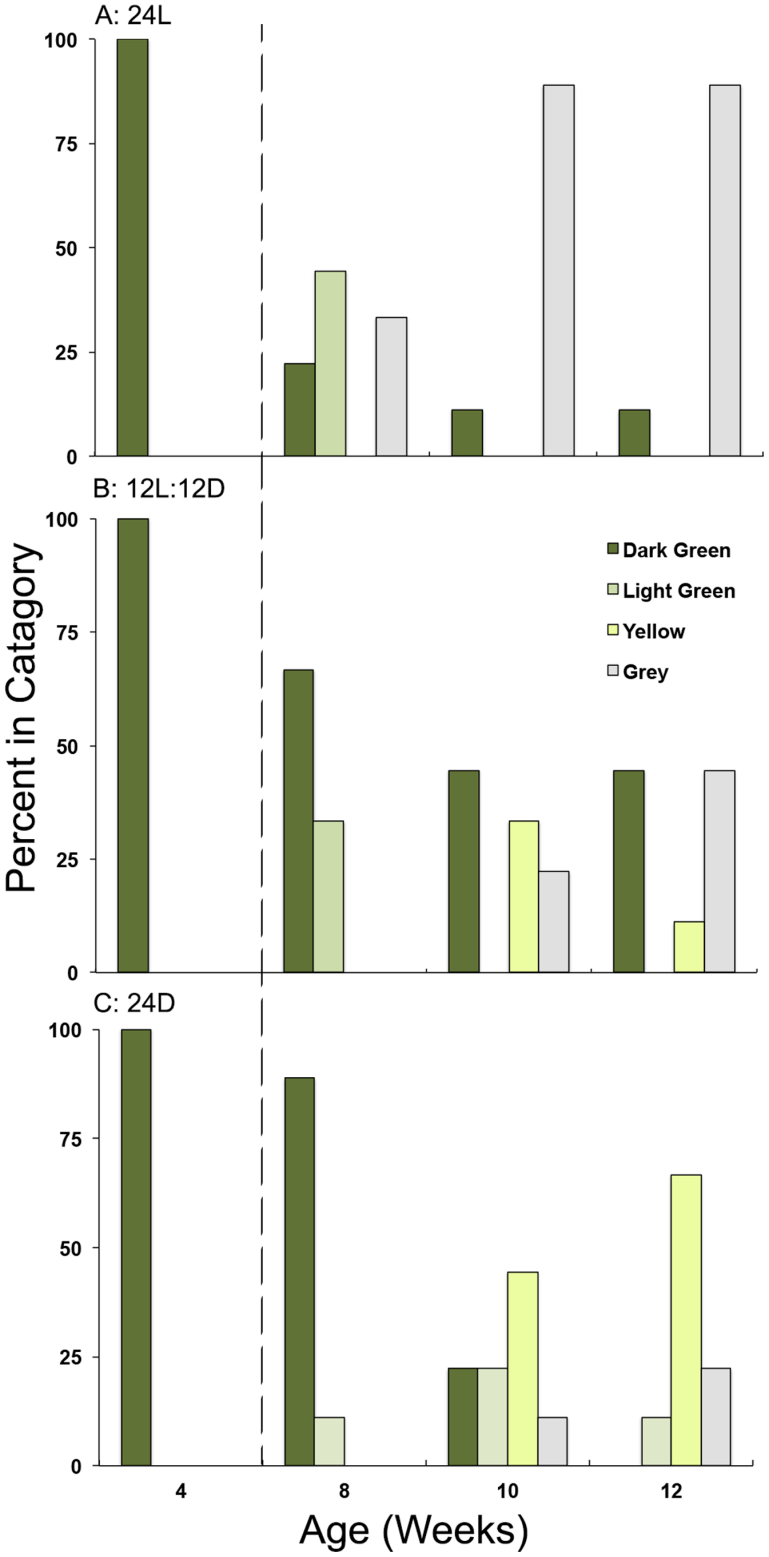
Representative pigment composition of animals that were raised in A) 24L, B) 12L∶12D, or C) 24D conditions and provided *V. litorea* for 4 wk before starvation for 4,6, and 8 wk. Animals were scored based on a color rubric ranging from dark green to grey, with movement away from dark green indicative of loss of plastids. There was a greater percentage of animals observed with pigment loss in the 24D (C) and 24L (A) conditions, than in 12L∶12D (B). Dotted line indicates when food was removed from all treatments.

Observations of pigment composition of these animals at each time point were ranked according to 4 levels: a healthy dark green animal, ranked 1, and represented a “natural” specimen akin to those observed the field; while a light grey animal lacking pigment altogether ranked 4. These pigment metrics (Figure 12) combined with the amount of mortality (Figure 11B) showed a clear negative effect of lack of light on *E. chlorotica* fitness. Animals maintained on a natural diurnal rhythm (12L∶12D) showed the fewest number of animals exhibiting pigment loss (Figure 12B), and healthy animals were observed throughout the starvation period (Figure 11B). Surprisingly, many animals exposed to 24L conditions also exhibited pigment loss after 6–8 wk of starvation (Figure 12A); however, there was no concurrent increase in mortality (Figure 11C). In animals maintained in the dark, all exhibited both a loss of pigment with starvation (Figure 12C) and concurrent high mortality rates (Figure 11B). Taken together, these data suggest that photosynthate is important in stabilizing the symbiosis in *E. chlorotica*.

## Discussion

Since the initial description of functional kleptoplasty in *Elysia* ( =  *Tridachia*) *crispata* by Trench [11], the mechanisms enabling these sacoglossans to maintain and protect plastids ‘stolen’ from their algal food have long remained a mystery. The processes involved in establishment of the symbiosis are finally being unraveled, thanks in part to the ability to sustainably rear the animals in the laboratory [13]. This has resulted in the first observations of initial plastid establishment within the digestive diverticula of *E. chlorotica*. What has emerged is the apparent importance of LDs in this symbiosis. From repeated observations of numerous individual *E. chlorotica* specimens using confocal and TEM, we can conclude that the large deposits observed are neutral lipids, although OsO_4_ counterstaining did not yield the typical optically opaque structures (Figure 3C,D). Rather, the lipids in *E. chlorotica* electron micrographs appear a uniform grey color. This staining has been seen historically in other work using TEM and *E. chlorotica* [2], [6], [8], [19], but not to the extent and abundance observed here. The LDs have also been loosely implicated with food availability [2], [6], [8], [19]. Unique to these specimens is the observation of lipids accumulating *within* the plastids in the animal. Such lipid accumulation has not been observed in plastids within *V. litorea* filaments [6], [19], [20]. This is not to say that the filaments of *V. litorea* do not accumulate lipids, in fact, *V. litorea* is a known lipid producer (see below) and lipids are the main storage product of the alga [21]. The obvious accumulation of lipids in conjunction with plastids (Figure 9), coupled with the decreased plastid stability, retention and animal survival when photosynthate is not produced (Figure 11), suggest a physiological role of the LDs in establishing and potentially maintaining plastid stability within *E. chlorotica*.

In the past 10 years, LDs have gone from being an overlooked depository to now being considered self-standing dynamic organelles, complete with enzymes, proteins and metabolic functions [22]–[25]. In addition, LDs are known to have a role in immunity and host-pathogen interactions [26]–[30]. In other symbiotic relationships, primarily the zooxanthellate symbioses, the role of lipids as metabolic currency has been suggested, but not yet clearly resolved [31]–[43] Outside of the pioneering work on plastid symbiosis in sacoglossans [11], [12], [44]–[46], much less is known about the transfer of photosynthate from kleptoplasts to their hosts, and little attention has been given to the role of lipids in sacoglossans. Trench et al. [46] identified radiolabeled ^14^C in both phosphatidylcholine and phophatidylglycerol isolated from *E. viridis* (which utilizes plastids from the chlorophyte alga *Codium*). This presumably resulted from labeled precursors exported from the plastids and subsequently incorporated into lipids by the animal. It is likely that in sacoglossan kleptoplasty, as in coral-zooxanthellate symbiosis, lipids may be provided to the host by the plastid.

### Lipid dynamics during plastid establishment

Observations of developing juveniles using TEM showed a clear correlation between plastid incorporation and lipid production. Images of aposymbiotic animals revealed no lipids, but within 24 h of feeding both plastids and lipids were present in the animal and accumulated rapidly as long as food was available. The effect of starvation depended upon the age/developmental stage of the animal. In animals fed for ≥7 d the majority of the micrographs observed contained clearly intact plastids and numerous lipid stores – which were not observed in animals fed 5 d and then starved. These microscopic observations support progression from transient to permanent kleptoplasty observed in developing juveniles between 5–7 dpm [13], and suggest the involvement of lipids in this shift. It is clear that there is an energetic relationship between the plastids of *V. litorea* in their animal host and the ability to establish permanent kleptoplasty.

Trench et al. [46] observed degradation of plastids surrounded by what appeared to be autosomal membranes in adult *E. crispata* starved for 30 d in the dark. Similar observations were made in juvenile *E. chlorotica* after only 3 d of starvation (albeit in the light; Figure 5B). It is apparent that *Elysia* sp. do have a mechanism for intracellular plastid breakdown, but this process may be inhibited by the plastids, or driven by plastid abundance. In contrast to the young developing juveniles, adult animals did not exhibit this rapid response to starvation at the cellular level. Adult animals that had been fed regularly in the lab for 11 months before a 2 wk starvation period showed no apparent increase in phagosome number or marked decrease in lipids or plastids (Figure 8).

To indirectly test the role of photosynthate in stabilizing the plastids in the host, animals were allowed to develop and feed for 1 month in either light (24L; 12L∶12D) or dark (24D) conditions. In this scenario, plastids were present in the animals, but only animals exposed to light were able to photosynthesize between feedings (2× weekly) and during starvation. Animals maintained in the dark were comparable in size and color to the animals in the light treatments for only their first week of development. This similarity suggests that the production of photosynthate may not be required to enable the initial plastid establishment, or that the inherent lipid content of the plastids was enough to enable the initial plastid establishment at 1 wk. After this initial week of feeding, differences between the treatment groups emerged. Animals maintained in the dark showed a loss in size, pigment and increased mortality; this too suggests a role of photosynthate in growth and/or maintenance. *E. chlorotica* maintained in either light condition still had healthy dark green specimens present after 8–12 wk starvation, and experienced little to no mortality over the duration of the experiment. Because inter-individual variability has been shown to be a factor when working with *E. chlorotica* [47], along with factors such as brood history and environmental conditions, these experiments were repeated twice with 12 and 20 animals in each treatment. In each case, >50% of the animals kept in the dark lost their plastids after a month of starvation compared to <10% in light treated animals (data not shown). Unlike recent data presented by Christa et al. [48] in which the two replicate animals of either *E. timida* or *Plakobranchus ocellatus* did not show any need for light for survival, the data presented here using 1 month old adult *E. chlorotica* do support the need for light-generated photosynthate. Without the production of photosynthate by the plastids, the symbiosis in *E. chlorotica* is much more tenuous with subsequent pigment loss, size loss, and mortality all being greater in animals maintained in dark *vs*. light conditions. The kleptoplasty observed in *E. chlorotica* is indeed unique from *E. timida*, *Plakobranchus ocellatus* and other sacoglossan sea slugs. That *V. litorea* is a lipid producing alga, the abundance of lipids observed, the dependence on light, and the inherent long-term stability of the plastids continue to distinguish this kleptoplasty from others.

### Plastids as the source of lipids

The observations made here present both visual and biochemical support for plastid derived lipids (or fatty acids) in the host, which may serve as a carbon reserve. In oleaginous algae (e.g., *V. litorea*), massive lipid production can be induced upon exposure to certain stressors such as nutrient deprivation or salinity fluctuations; this is an area of particular interest for algal biofuel production [49]–[53]. Indeed, as research in algal lipid production has progressed, it appears that lipid synthesis in algae is distinct from that of Viridiplantae. Even within algal groups, there is a considerable amount of individuality in terms of the lipids produced and the biosynthetic mechanisms used [54]–[57].

One of the greater distinctions in algal lipid production is the ability of certain algal plastids to produce TAGs *de novo* within the plastid prior to export [58], [59]. The appearance of lipids within the *Vaucheria* plastids in the animal (Figure 9), but not in the algal filament [6], [19], [20], supports the possibility of stressed conditions (presumably from being ingested and phagocytosed by *E. chlorotica*) triggering the spontaneous production of lipids in the *V. litorea* plastid. Further experimentation will be needed to verify the types of lipids observed in these plastids and if the LDs observed in the host do indeed originate in the “stolen plastids.”

Fatty acid analysis of the animals and alga did provide support that the alga is contributing directly to the fatty acid profile of the host. Certain fatty acids can be synthesized and utilized by the both the alga and animal, however, EPA (20∶5) is a PUFA produced primarily by marine algae, fungi and some microbes [60]. This PUFA is accumulated in animals via food intake and cannot be synthesized by any animals known to date. Thus, its presence in such high abundance in *E. chlorotica* supports that fatty acids in the animal may originate from the algal plastids (Figure 10C).

### Putative mechanism of plastid stability

The data presented here support that the lipids produced by *V. litorea* are involved in establishing or stabilizing kleptoplasty in *E. chlorotica*. The simplest explanation of this relationship is an energetic one. The *de novo* synthesis of lipids by the plastids providing the host with an energy reserve is analogous to what has been suggested in the zooxanthellate symbiosis [39]–[43]. *V. litorea* naturally produces lipids as an energy source. In *E. chlorotica*, plastids incorporated in massive numbers contribute all the visible green pigment to the animal. When light is available, photosynthate continues to accumulate in the animal in the form of LDs. These lipids are then presumably used as an energy source by the animal, leaving the plastids undisturbed in the animal cytosol. If lipid stores are depleted, plastids are targeted for breakdown by the animal as was observed in the animals exhibiting transient kleptoplasty (Figure 5). Conversely, once lipids accumulate in the animal to a level that sustains metabolic need, the plastids are not broken down by the host and permanent kleptoplasty is acquired. Without lipid accumulation, plastids can be initially acquired but are not stabilized, as seen in animals maintained in dark conditions that suffered detrimental effects including pigment loss, loss of size and increased mortality (Figure 11).

In addition to serving as an energy bank, lipids may also play a direct role in protecting the plastids and contributing to their long-term stability through two possible mechanisms: 1) physically protecting the plastids and/or 2) mediating the host immune response. Lipids have been shown to physically protect plastids when introduced into protoplasts, and uptake efficiency, pigment content and oxygen evolution rates of liposome-treated plastids were higher relative to controls [61]. Additionally, n-3 PUFAs have recently been shown to function as antioxidants [62], [63], potentially through mediating gene expression of antioxidative enzymes [64]. Protective antioxidative mechanisms would have a direct positive effect on the intracellular environment of the plastids, where photooxidative stress would presumably generate large amounts of reactive oxygen species. The abundance and intimate location of the LDs relative to the plastids in developing *E. chlorotica* (e.g., Figure 6G,I), suggest a protective mechanism that remains to be fully characterized.

In addition to the inherent protective nature of the LDs, the precise timing of the change from transient to permanent kleptoplasty (between 5–7 dpm) suggests a host-derived phenomenon may also be involved in stabilizing the plastids. Such a change in *E. chlorotica* might involve suppression of the host immune response, of phagosomal maturation or of autophagy, all of which would elongate the intracellular plastid presence. Lipids accumulate in response to several pathogenic infections [28], [65]–[68], analogous to what was observed in response to ‘infection’ by the *V. litorea* plastids. LDs can directly mediate phagolysosome maturation in certain infections [29], [66], [69], [70], as well as directly affect autophagy-related processes [71]–[73]. The n-3 long chain PUFAs (such as EPA) modulate immune responses in a wide variety of *in vivo* and *in vitro* studies in numerous organisms reviewed by [74]–[76]. PUFAs (such as EPA) can affect numerous aspects of the host immune response, which would directly benefit plastid function in *E. chlorotica* including components of the innate immune response such as inflammation [77], superoxide production [78], phagocytosis [79], and peroxide production [80]. High amounts of such PUFAs have a negative effect on the ability of hosts to clear pathogen infection [75], [81]–[86]. The establishment of the plastids within the digestive diverticula of *E. chlorotica* is in many ways analogous to an infection. If these lipids suppress the ability of the host to clear the plastids, this would ultimately aid in the intracellular establishment of the foreign organelles.

## Conclusions

LDs from *V. litorea* plastids are transferred to the host where they play a critical role in the initial and long term stability and functionality of the kleptoplastic relationship. The presence or abundance of plastids alone is not enough to sustain the symbiosis; rather, it is the photosynthetic ability of these plastids in the animal. LDs, presumably a result of photosynthesis, serve to stabilize the plastids in the host, enabling progression towards permanent kleptoplasty. How these lipids perform this task, albeit in the form of energy for metabolism, through physical protection, or through modulating host immune responses are current areas of investigation. It is apparent that LDs play a pivotal role in the *E. chlorotica – V. litorea* symbiosis as has been observed in numerous other symbioses and pathogenic infections.
